# Atomization characteristics and instabilities in the combustion of multi-component fuel droplets with high volatility differential

**DOI:** 10.1038/s41598-017-09663-7

**Published:** 2017-08-21

**Authors:** D. Chaitanya Kumar Rao, Srinibas Karmakar, Saptarshi Basu

**Affiliations:** 1 Department of Aerospace Engineering, Indian Institute of Technology Kharagpur, Kharagpur, 721302 India; 2 Department of Mechanical Engineering, Indian Institute of Science, Bangalore, 560012 India

## Abstract

We delineate and examine the successive stages of ligament-mediated atomization of burning multi-component fuel droplets. Time-resolved high-speed imaging experiments are performed with fuel blends (butanol/Jet A-1 and ethanol/Jet A-1) comprising wide volatility differential, which undergo distinct modes of secondary atomization. Upon the breakup of vapor bubble, depending on the aspect ratio, ligaments grow and break into well-defined (size) droplets for each mode of atomization. The breakup modes either induce mild/intense oscillations on the droplet or completely disintegrate the droplet (micro-explosion). For the blends with a relatively low volatility difference between the components, only bubble expansion contributes to the micro-explosion. In contrast, for blends with high volatility differential, both bubble growth as well as the instability at the interface contribute towards droplet breakup. The wrinkling pattern at the vapor-liquid interface suggests that a Rayleigh-Taylor type of instability triggered at the interface further expedites the droplet breakup.

## Introduction

Effective liquid atomization is crucial in a wide range of engineering applications such as spray combustion in automotive and gas turbine engines, powder technology, spray drying, spray cooling and ink-jet printing. The fuel atomization characteristics in engine applications are crucial in determining combustion stability, efficiency, and exhaust gas emissions. In order to estimate these vital characteristic features of an engine, it is necessary to have a clear understanding of the spray structure, in particular, the spatio-temporal distribution of droplets. In a conventional fuel spray, the dense liquid columns/sheets are prone to instabilities due to the aerodynamic interactions, which lead to the formation of ligaments. These ligaments further breakup into droplets. The first generation droplets further undergo breakup to form smaller sized daughter droplets (secondary atomization) which subsequently undergo evaporation and combustion. Secondary atomization of emulsions and multi-component fuel droplets plays an active role in minimizing CO_2_, NO*x*, and unburned soot particles emanating from the combustion process. The combustion efficiency and exhaust gas emissions can also be improved by using biofuels as additives to conventional transportation fuels. Oxygenated biofuels such as ethanol and butanol are extensively used as additives to gasoline/diesel to improve engine performance and reduce the harmful emissions^1, 2^. In particular, Maurya *et al*.^2^ reported that the maximum combustion efficiency achieved for HCCI engine fuelled with ethanol is around 97.5% while the NO*x* emissions from the engine were found to be extremely Low (<10 ppm). Ethanol or butanol can be also be added to aviation-grade kerosene for aero-propulsion applications. However, the burning question remains, how would the combustor design be adapted to make them fuel flexible. In general, alcohol blended fuels have the potential to improve the atomization and subsequent combustion in diesel engines and gas turbine combustors. However, fundamental research pertaining to atomization and combustion characteristics is extremely rare, especially at the droplet level. This is even more important considering the fact that droplets actually form the sub-grid level of any evaporating/combusting fuel spray. It is our effort to provide some key physical insights into blended fuel droplet combustion so that later these results can be ported to aid in combustor design without significant trial and error. In addition, the current work will also aid in understanding and alleviating phenomena like combustion instabilities in engines and turbines. It is expected that combustion instabilities in such systems will originate from inadequate atomization, vaporization and mixing at the droplet level and cause extreme loss of performance and efficiency. It is hence important to characterize such effects in controlled experiments using different levels of alcohol blending. An emulsified or a multi-component fuel droplet, comprising of two or more liquids with significant volatility differential can undergo rapid disintegration^3–14^. Due to the distinct differences in boiling points, the higher volatile constituent can be superheated, leading to the formation of a critical-sized vapor embryo by spontaneous homogeneous nucleation. The explosive growth of this vapor bubble causes fragmentation of liquid droplet into small secondary droplets. During the combustion of multi-component fuel droplets, the volatility differential, the relative concentration of fuel components as well as size and location of the bubble at the onset of breakup controls the intensity of droplet disintegration^5, 8, 9^. The violent rupture of vapor bubbles causes surface dynamics like interface corrugations in droplets, which may either lead to complete breakup of parent droplet (micro-explosion) or induce non-periodic shape oscillations. Understanding these breakup phenomena and the resulting shape deformations in the combustion of multi-component fuel/emulsion droplets can be advantageous in improving fuel efficiency in spray combustors. Several studies were performed on fragmentation and shape deformation of droplets through collision with solid surfaces and by severe aerodynamic loading^15–25^. Extensive experimental investigations on the atomization of bi-component droplets as well as nano-particle laden droplets were studied under the influence of acoustic field^26–34^. Recently, Miglani *et al*.^29, 30^ described different modes of ligament breakup and the subsequent volumetric shape oscillations of the parent droplet during the combustion of nanoparticle-laden high vapor pressure fuel droplets. The deformation in the droplet geometry was quantified using an ejection impact parameter (α). In addition, Landau-Darrieus (LD) evaporative instability was suggested to cause interfacial instability at the vapor-liquid interface. A similar observation was made by Shepherd *et al*.^35^ during the growth of a bubble inside a butane droplet at superheat limit. During atomization of liquid in high-speed gas flows, shear induced capillary waves grow at the vapor-liquid interface (Kelvin-Helmholtz instability) which may lead to the growth and breakup of the ligaments^36^. In addition, the density difference across the vapor-liquid interface drives Rayleigh-Taylor (RT) instability^37–40^, which may augment the fragmentation of droplet. RT instability has been reported in a few experimental investigations on the rapid evaporation of liquids at superheat limit^35, 41–43^ as well as in numerical investigations^44–47^. Elgowainy *et al*.^45^ employed a mathematical model and suggested that RT instability controls the hydrodynamics of later stage of bubble growth and affects disruptive phenomenon as well as the droplet breakup time in an emulsified fuel. In our recent studies^48, 49^, the micro-explosion characteristics of butanol/Jet A-1 and ethanol/Jet A-1 were examined to understand the nucleation, bubble growth, and flame disruption due to micro-explosion; however, no analyses were performed to gain quantitative insights on instability mechanisms occurring at multi spatio-temporal scales.

The present work is aimed at understanding the mechanisms responsible for the dynamics of droplet breakup and instabilities in the combustion of multi-component fuel droplets. The variations in ligament formation, growth, and breakup into droplets (i.e., the secondary breakup pathway) are studied for different fuels blends. We mainly focus on two modes of instabilities. First, we investigate the modes of atomization induced by the breakup of vapor bubble and the subsequent shape oscillations in the parent droplet. The second part of this study is focused on the interfacial perturbation and droplet shape oscillations induced by the Rayleigh-Taylor instability at the vapor-liquid interface and its resulting effect on the droplet breakup. The results presented here support the proposition of Rayleigh-Taylor instability during the combustion of droplets with significant volatility differential. This study provides first such experimental evidence of wrinkling pattern and surface undulations caused by RT instability in the context of multi-component fuel droplets. In essence, the primary objective of this study can be categorized into three sections viz; (1) the ligament breakup mechanisms in different fuel blends, (2) shape oscillations and (3) interfacial instabilities during the lifetime of a single fuel droplet.

## Results and Discussion

**Figure 1:**
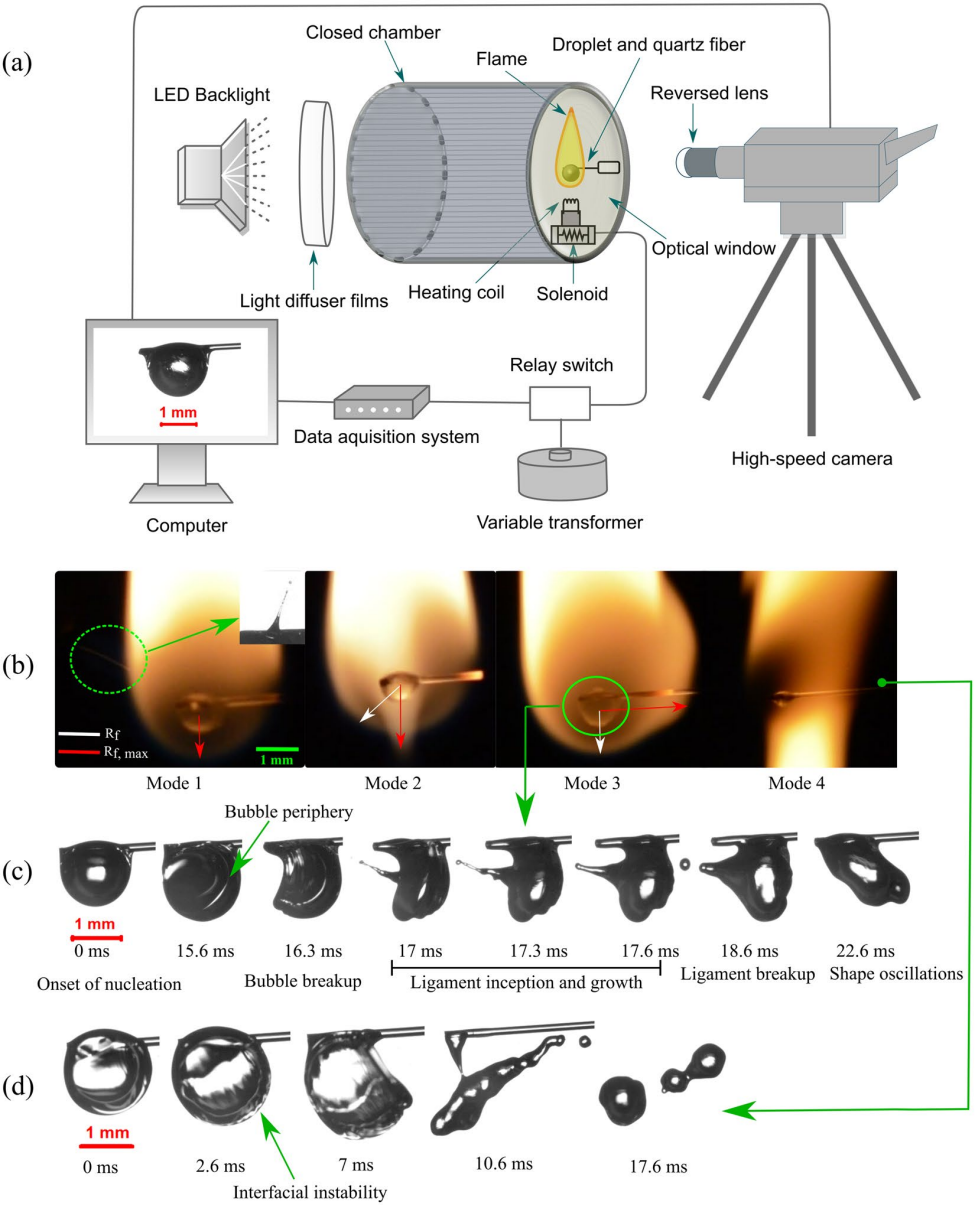
(**a**) Schematic of the experimental apparatus. Schematic of various types of droplet breakup. (**b**) Modes of ligament breakup; (**c**) A typical temporal sequence of Mode 3 type bubble breakup, ligament growth, ligament breakup and subsequent shape oscillations; (**d**) Micro-explosion due to both internal expansion and interfacial instability.

The combustion of multi-component fuel droplets with wide volatility differential is expected to induce perturbations at varying length scales on droplet surface due to the breakup of vapor bubble and consequent ejection of secondary droplets. The growth of vapor bubble may lead to either puffing (partial breakup) or micro-explosion (complete disintegration) of the parent droplet. A multi-component or emulsion fuel droplet can also undergo sudden disintegration (recognized as abrupt explosion) without any visible bubble growth^49^. Breakup due to bubble growth is comparatively higher in droplets with larger proportion of volatile components (30% and 50% v/v blends). Since only ligament mediated breakup is studied in the present work, abrupt explosion is not considered here. Similarly, in case of butanol blends, nucleation does not take place in B10 blends^48^. Therefore, the breakup characteristics are studied for B30 and B50 blends. The volatility difference between ethanol and Jet A-1 is comparatively higher than that of butanol and Jet A-1. A typical bubble breakup event involves bubble collapse, ligament development, and finally ligament fragmentation to form secondary droplets. Most of the ligaments undergo pinch-off while a few ligaments grow and retract to the liquid surface. The pinched-off secondary droplets are subsequently transported to the surrounding flame since the ligament possesses an inherent outward momentum after bubble breakup. Based on the secondary droplet expulsion intensity, the secondary droplets may either evaporate promptly at the flame zone (minor breakup events) or lead to complete flame disruption due to micro-explosion. Thus, it is important to understand the influence of distinct atomization modes prevalent at different proportions of volatile component on the flame disruptive behavior. Based on the augmentation in the severeness of droplet deformation (Fig. 1), the bubble breakup events can be referred to as low-intensity minor breakups (Mode 1); intermediate intensity breakups (Mode 2); high-intensity major breakups (Mode 3); and micro-explosions (Mode 4). A minor breakup event usually indicates ejection of small-sized secondary droplets, which initiates small-scale surface roughening and moderate oscillations in droplet volume. Similarly, a major breakup event indicates major bubble collapse event that corresponds to the breakup of a substantial bubble volume leading to severe droplet deformation. Besides, the parent droplet may not recover from the high degree of deformation induced by the fragmentation of a significantly large bubble leading to micro-explosion.

Figure 1a illustrates the schematic of the experimental setup. Different modes of breakup and the subsequent distortion in the droplet flame is shown in Fig. 1b, where R_f_ indicates the radius of the bottom hemisphere of the flame and R_f,max_ represents the maximum distorted flame radius after the disruptive phenomena. The faint flame streak (inside a dotted circle) represents mode 1 type atomization, while the inset shows the representative magnified image of the mode 1 type ligament. Unlike mode 1 type atomization, mode 2 type breakup causes small scale distortion in the flame. Figure 1c depicts a sequential pathway of mode 3 type breakup consisting of bubble breakup, ligament growth, ligament breakup, and finally, breakup induced droplet shape oscillations. Similarly, the complete disintegration of the droplet (mode 4) is also represented in Fig. 1d. Mode 4 type atomization accounts for the formation and breakup of thick ligaments along with instability in the precursor stages of droplet breakup. The detailed description of different modes of atomization will be discussed in the next section.

The degree of breakup is primarily dependent on the size of the bubble inside the parent droplet, and the relative ratio of vapor bubble volume to droplet volume at the pre-breakup instant^29, 46^. A breakup impact parameter^29^ is employed to quantify different modes and intensity of breakup, which is defined as

1 $${\alpha }_{global}(t)=(\sum _{j=1}^{n}\frac{\pi }{6}{d}_{j,breakup}^{3})/(\frac{\pi }{6}{D}_{breakup}^{3}),$$

where *D* denotes the diameter of parent droplet at the onset of homogeneous nucleation of vapor bubble and *dj* denotes the diameter of the *j*th bubble at the pre-breakup instant. Here, numerator indicates the bubble volume, which either collapses in the droplet interior or completely disintegrates the parent droplet during *n* simultaneous breakup events. In a nutshell, the breakup impact parameter, *α*_*global*_ (t) signifies the degree of breakup that estimates the level of deformity in the droplet geometry following the breakup of *n* vapor bubbles. A related parameter *α*_*local*_ (*t*) indicates the deformation of parent droplet for a single breakup event, which is defined as

2 $${\alpha }_{local}(t)=(\frac{\pi }{6}{d}_{breakup}^{3})/(\frac{\pi }{6}{D}_{breakup}^{3}),$$

Combustion of miscible multi-component fuel droplets usually involves growth and rupture of a single bubble at a particular instant; therefore, *α*_*local*_ is used as a deformation index in the present work to characterize the ligament dynamics and subsequent shape oscillations induced on the parent droplet as discussed in the following sections.

### Ligament dynamics

Bubble breakup leads to the development of a crater on the droplet surface. The formation of the crater and the ensuing pressure difference results in the inception of a ligament, which grows and breaks apart. The observed ligament growth and consequent pinch-off are analogous to a typical pinch-off arising from the capillary instability, indicating that the evolution of the ligament on the droplet surface is primarily governed by the competitive effects of surface tension force and ligament inertia while viscosity plays a stabilizing role. Besides, the surface tension acts in two different ways on a developing ligament. Surface tension along the ligament length tries to inhibit ligament stretching. The surface tension force along the circumference aids in the reduction of neck radius resulting in droplet pinch-off. Two general modes of pinch-off observed in the present study are the long-wave and short-wave modes. It is important to note that these modes are similar to the ones found in the breakup of ligaments during the atomization of a conventional fuel spray. The long-wave mode involves development and breakup of high aspect ratio (ligament length/diameter) ligaments (~10–15) which is driven by short period, fast capillary waves. In contrast, the evolution of short-wave breakup mode is slow, and the pinch-off of thick ligaments occurs successively from the tip. The modes of ligament breakup are further characterized based on the breakup impact parameter (*α*). The different modes are then correlated to the ligament aspect ratio and size of the pinched-off secondary droplet. The ligament breakup modes are categorized as: (1) high momentum needle-type ligaments with both tip-base breakup (*α* < 0.1); (2) low momentum needle-type ligaments with tip breakup, tip-base breakup, and multiple sequential breakups (0.1 < *α* < 0.5); (3) low momentum, thick ligaments with tip breakup, tip-base breakup, and multiple sequential breakups (0.5 < *α* < 2); (4) very low momentum, thick ligament growth and breakup due to micro-explosion (2 < *α* < 5).

#### Mode 1 type breakup

**Figure 2:**
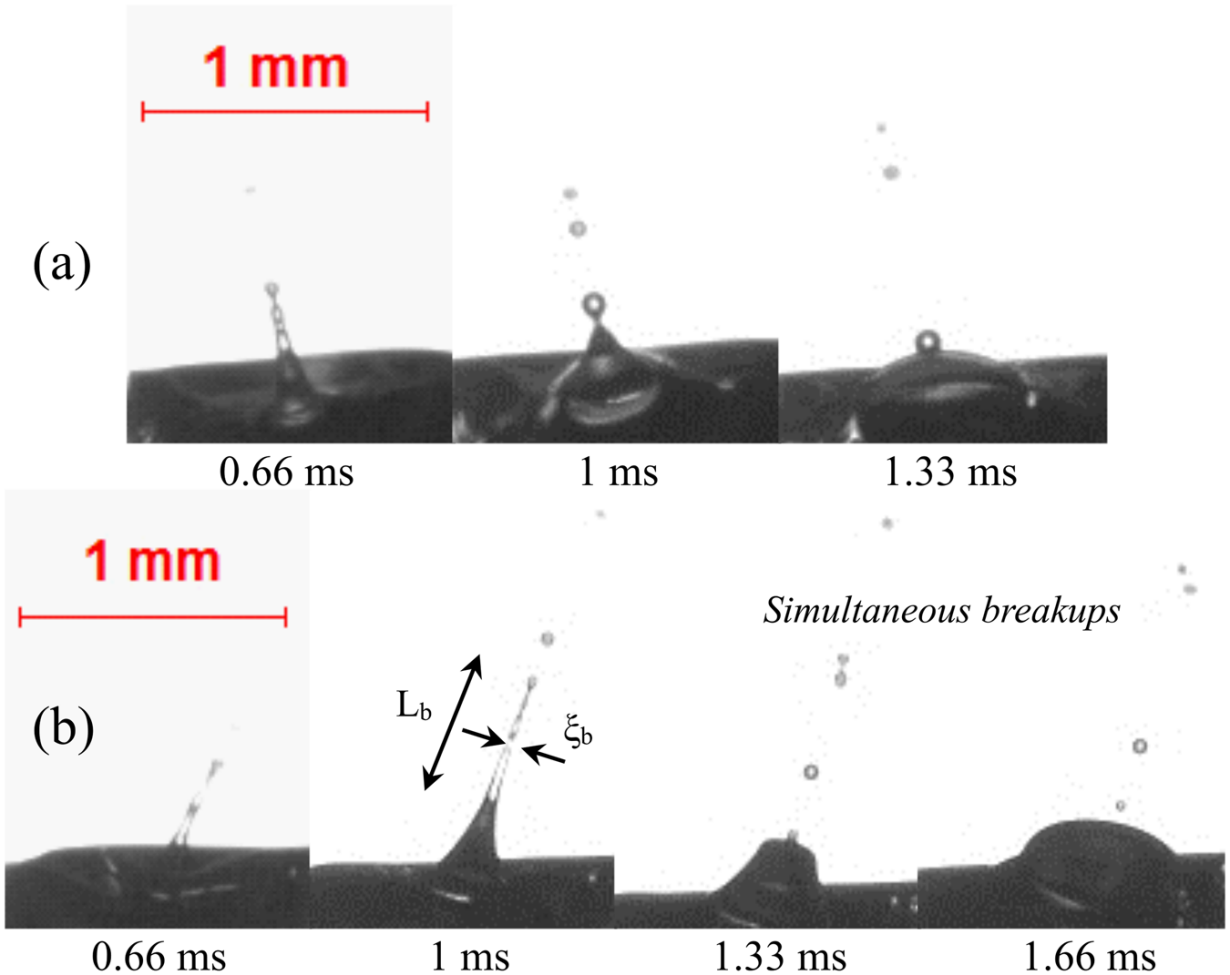
High aspect ratio, needle type ligament growth and breakup (Mode 1) for (**a**) α = 0.02, AR = 10.8 and (**b**) α = 0.05, AR = 15, representing simultaneous necking and pinch-off of secondary droplets in a typical E10 and B30 droplets. Both the ligament and the ejected secondary droplets carry high momentum (max. velocity ~2 m/s) upon the breakup of the bubble.

Figure 2a represents mode 1 type ligament breakup which is primarily characterized by the breakup of cylindrical, high aspect ratio ligaments (~10–15). The ligament length L_b_ and diameter ξ_b_ of the unperturbed ligaments are measured from the high-speed images. It is noticeable in Fig. 2a and b that the secondary droplets breakup almost instantaneously along the entire ligament length, thus forming multiple fine secondary droplets. Mode 1 type ligaments originate due to the breakup of small sized bubbles (*α* < 0.1). Since the pressure inside a smaller bubble is usually higher, upon breakup, the pressure pulse is converted into kinetic energy of the ligament and pinched off daughter droplets. This indicates that the high aspect ratio ligaments have the tendency to collapse early and exhibit larger momentum than that of lower aspect ratio ligaments^30^. The pinched-off secondary droplets possess a significant outward momentum (max. velocity ~2 m/s). Mode 1 type ligament breakup events occur at relatively short time scales τ_eject_ ~ O (1.3 ms). τ_eject_ is the total elapsed time from the onset of bubble breakup till the formation of the final secondary droplet. The typical ligament breakup timescale (τ_b_) of Mode 1 is O (0.6 ms), which is the time elapsed from the formation of ligament to the generation of the final secondary droplet. The maximum diameter of the pinched-off secondary droplet in this mode is O (0.05 D_0_). Overall, the probability of this mode is very low, and it is mostly observed in E10 and B30 blends, which can be attributed to the formation of relatively fewer nucleation sites and subsequent breakup of small bubbles.

#### Mode 2 type breakup

**Figure 3:**
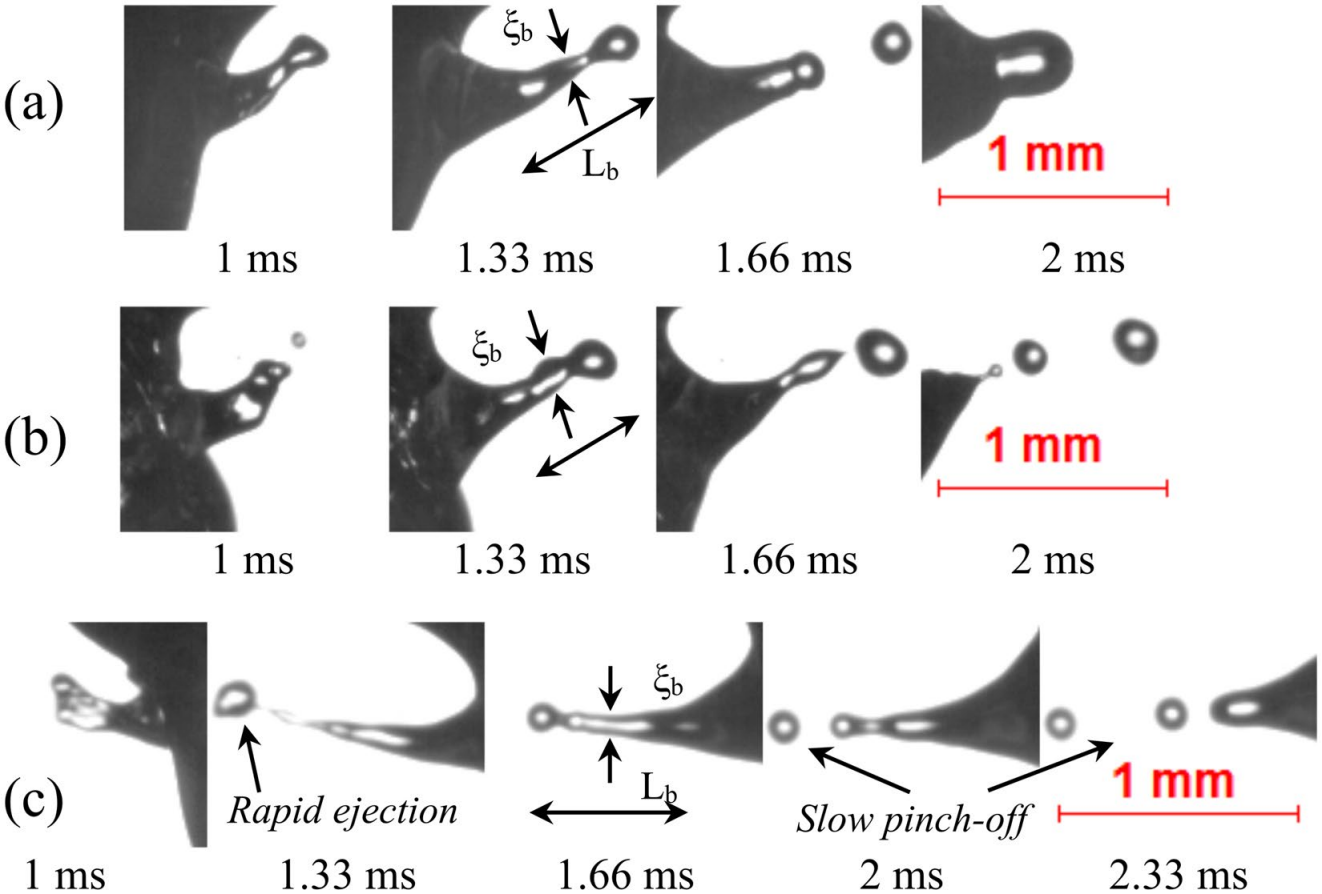
Low momentum ligament growth with (**a**) tip breakup (α = 0.2, AR = 6.8) in B30 droplet, (**b**) tip-base breakup (α = 0.2, AR = 3.96) in E30 droplet, and (**c**) formation of multiple secondary droplets in E30 droplet (α = 0.43, AR = 5.7). The maximum velocity of the secondary droplets is O (1 m/s).

Mode 2 type ligaments are relatively lower aspect ratio ligaments (~4–7) whose breakup occurs at a slightly larger timescale τ_eject_ ~ O (1.6–2.3 ms) than that of mode 1 type ligaments. The ligament possesses low momentum (max. velocity ~1 m/s), and its breakup can result in the formation of multiple secondary droplets. Depending on the length of the ligament, the secondary droplets can be generated either from tip breakup, tip-base breakup, or multiple ligament breakups. Figure 3a represents tip breakup of the ligament, which leads to the formation of a single secondary droplet. At τ_eject_ = 1 ms, the tip bulb pulls the ligament towards the axial direction due to surface tension. At this instant, the tip bulb pressure is high. The tip bulb subsequently becomes larger due to contraction. At 1.33 ms, the tip bulb pressure is less than the pressure at 1 ms. After the neck is developed, the pressure becomes high at the neck, since the tip bulb draws the liquid from the parent droplet. Finally, at 1.66 ms, when the neck becomes sufficiently thin (high pressure) pinch-off occurs (Fig. 3a). This mechanism of pinch-off is similar to the short-wave mode observed in a typical spray^36^. Soon after the formation of the secondary droplet, the remaining ligament retracts to the parent droplet. Similarly, a longer ligament breaks apart into 2–3 secondary droplets as seen in Fig. 3b and c. For a low momentum ligament with tip-base breakup, the total ejection timescale, τ_eject_ ~ O (2 ms). In contrast, for a ligament breaking into 3 secondary droplets, τ_eject_ scales as O (2.3 ms). The maximum diameter of the secondary droplet originating from a low momentum ligament breakup is O (0.1 D_0_). Mode 2 type ligament breakup is dominant in blends with 30% volatile component (B30 and E30). The overall probability of the occurrence of this mode is greater than that of Mode 1.

#### Mode 3 type breakup

**Figure 4:**
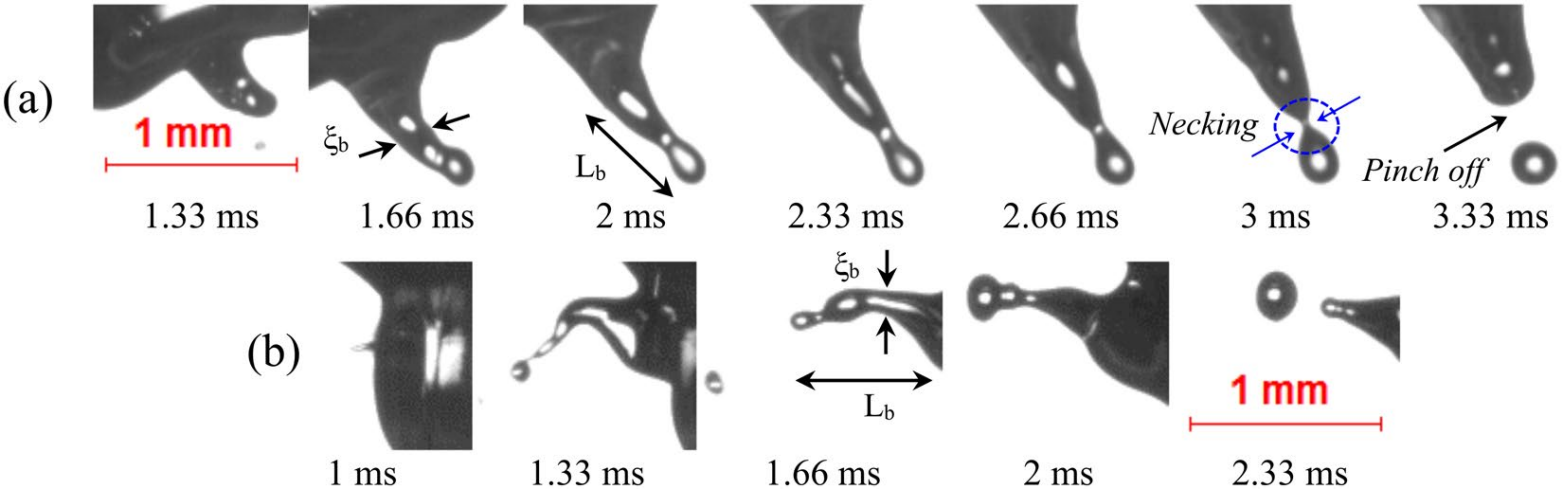
Low momentum thick ligaments with (**a**) tip breakup (α = 0.5, AR = 3.65) in E30 droplet and (**b**) tip-base breakup (α = 0.8, AR = 3.93) in B50 droplet. The maximum velocity of the pinched off secondary droplets is O (0.5 m/s).

Mode 3 represents breakup of slow evolving thick ligaments with an aspect ratio ~ O (3–4), leading to the formation of secondary droplets whose maximum velocity is O (0.5 m/s). The increase in the proportion of volatile constituent to 50% v/v results in the formation and breakup of thicker ligaments. This implies that more nucleation sites help in the coalescence of bubbles leading to the formation of bigger bubble. It can be observed in Fig. 4a that the ligament tip starts to grow at τ_eject_ = 1.66 ms. The tip bulb grows with time as it pulls the liquid by contraction. The growth of tip reduces the internal pressure and contracts the neck region of the ligament. Once the neck region becomes sufficiently thin, the circumferential surface tension force pinches off daughter droplet (at 3.33 ms). The timescale of this mode of ligament breakup is O (2.3–3.3 ms), and the maximum diameter of the ejected secondary droplet is O (0.13 D_0_). Mode 3 type breakup leads to large-scale surface undulations and translational motions of the droplet centroid (which will be discussed in the next section). Similarly, in the case of tip-base breakup (Fig. 4b), the ligament breakdowns sequentially into secondary droplets following tip contraction and necking. Mode 3 type breakup is predominant in E30 and B50 blends. It is evident that despite having a relatively small proportion of volatile component, ethanol blends undergo disruptive behavior more frequently than the butanol counterpart for the same blending ratio.

#### Mode 4 type breakup

**Figure 5:**
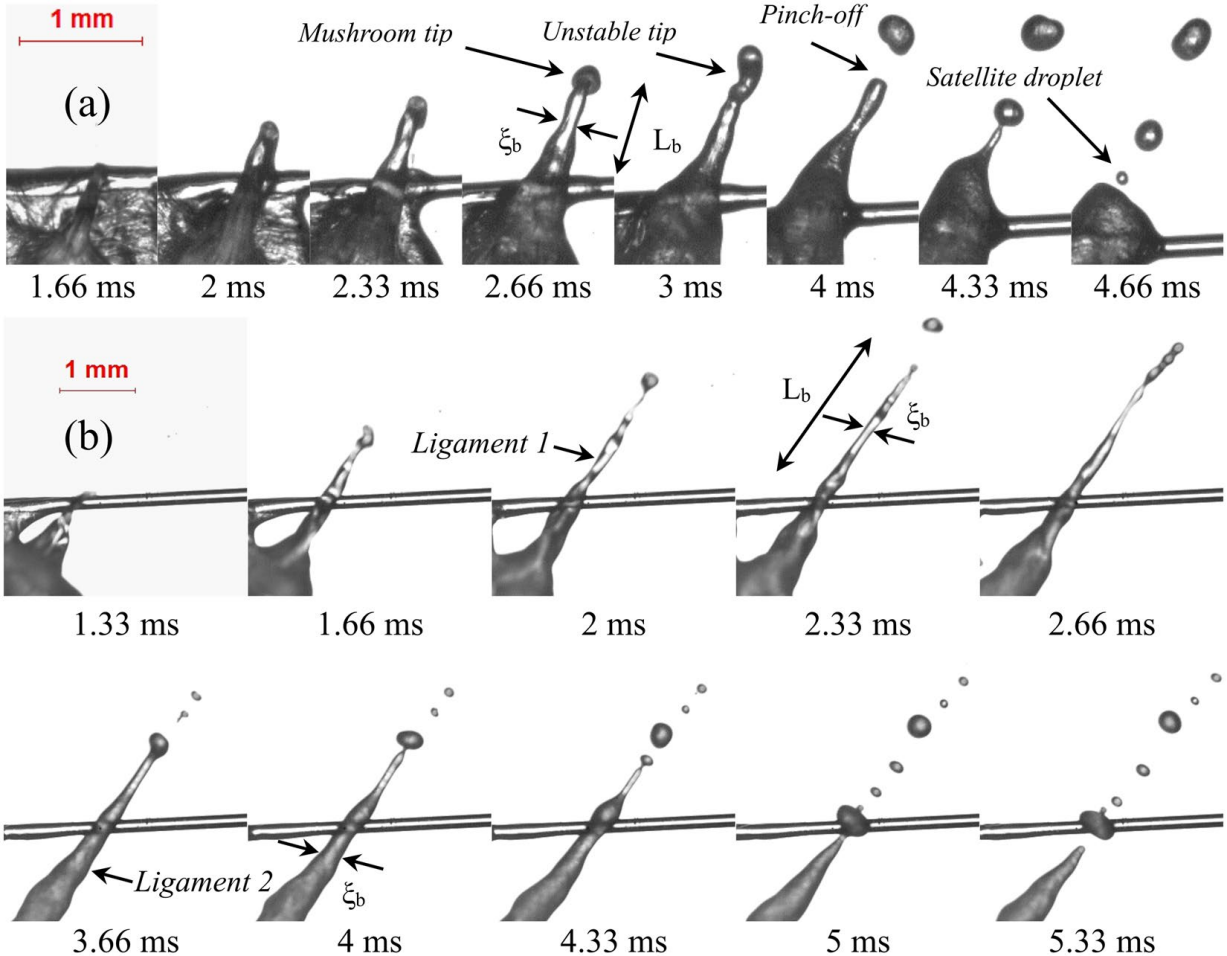
Ligament growth and breakup upon micro-explosion or complete disintegration of E50 droplets with (**a**) α = 3.2, AR = 3.72 (**b**) α = 5, AR = 17.

Mode 4 type ligament breakup occurs at the ejection timescale, τ_eject_ ~ O (4–6 ms). This breakup mode is predominant for fuel droplets with both larger volatility difference (Ethanol/Jet A-1 blend) and a greater proportion of volatile constituent (50% v/v). The breakup of this ligament results in the complete disintegration of the parent droplet. Moreover, the breakup of the bubble can lead to the formation of both thick (low aspect ratio) and thin (high aspect ratio) ligaments shown in Fig. 5a and b respectively, which further breaks up into large-sized multiple secondary droplets O (0.25 D_0)_. During the growth of thick ligaments, the tip of the ligament rolls up in a mushroom shape as seen in Fig. 5a. The mushroom-shaped tip is formed owing to the Rayleigh-Taylor instability as the ligament tip spreads in the lateral direction^36^. New secondary droplets are formed sequentially from the ligament along with satellite drops as seen in Fig. 5a. Apart from the formation of thick ligaments, high aspect ratio ligaments can also be seen if the intensity of breakup is much greater (α ~ 5) as seen in Fig. 5b. It is also evident that the breakup impact parameter predicts the intensity of ligament breakup with reasonable accuracy. Upon micro-explosion, multiple ligaments with different diameters are formed, which breakup into secondary droplets of distinct sizes. It is important to note that B50 droplets also undergo micro-explosion; however, the breakup does not result in the formation of a crater on the liquid surface to initiate ligament formation. Instead, the bubble breaks down in such a way that the air-liquid interface is disintegrated^46^.

#### General conclusions

**Figure 6:**
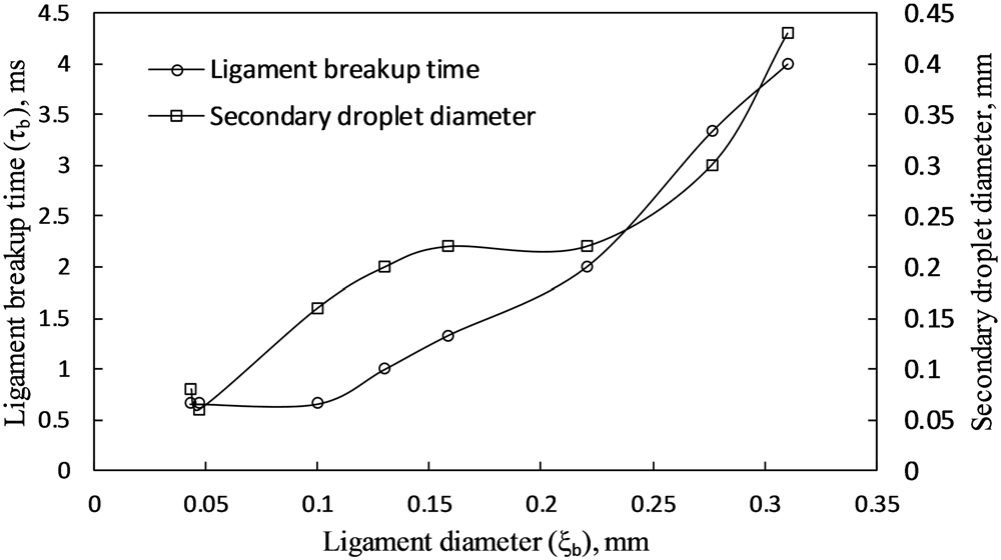
Variation in ligament breakup time and secondary droplet diameter following the breakup of ligaments of different diameters.

The low-intensity breakup modes 1 and 2 are predominant for blends with relatively lower proportions of volatile components. In particular, mode 1 is prominent for E10 and B30 blends and mode 2 is prominent for E30 and B30. Mode 3 type breakup dominates in E30 and B50 blends whereas mode 4 is dominant in E50 only. It is evident that for the same proportion of volatile component present in the blends, ethanol blends exhibit higher intensity breakup modes compared to butanol blends. The variation in ligament breakup time and the diameter of created secondary droplets for ligaments of different diameter is shown in Fig. 6. As the ligament diameter increases, the subsequent secondary droplet diameter as well as the total breakup time of each ligament increases. A similar observation was reported for the ligament-mediated breakup in nano-particle laden droplets.^30^ It is also apparent from the figure that the ligament breakup by capillary pinching produces secondary droplets of a size comparable to the ligament diameter.

The relative magnitude of viscous forces compared to inertial and surface tension forces is estimated by the Ohnesorge number, $oh=\mu /\sqrt{\rho \sigma {\xi }_{b}}$. It is important to note here that the Ohnesorge number (when the ligament erupts out of the crater) is well below unity for all blends (<0.1), signifying a predominance of capillary-inertial forces over viscosity of the liquid. Similarly, the relative influence of gravity on the inertial forces (estimated by the Froude number) is also smaller than unity for all the experimental test cases. The capillary time, which denotes the characteristic time of a capillary driven motion is given as:

3 $${\tau }_{c}=\sqrt{\frac{{\rho }_{L}{\xi }_{b}^{3}}{\sigma }},$$

where *ρ*_*L*_ is liquid density, σ is surface tension, and *ξ*_*b*_ is the ligament diameter. The predictions of the capillary time are consistent with the experimental ligament breakup time; however, the pre-factors are not (see Table 1). Similar to the breakup time, the ligament initiation time is also low when the aspect ratio of the ligament is large.

**Table 1:** Comparison of ligament breakup time with capillary time.

Aspect Ratio (AR)	Ligament initiation time, τ_eject -_ τ_b_ (ms)	Ligament breakup time, τ_b_ (ms)	Capillary time, τ_c_ (ms)	Pre-factor, (τ_b_/τ_c_)
3.65	1.33	2	0.6	3.33
3.72	1.66	3.33	0.855	3.89
3.93	1	1.33	0.37	3.59
3.96	1	1	0.276	3.62
5.7	1.33	1	0.264	3.78
6.8	1	0.66	0.25	3.55
10.8	0.66	0.33	0.06	5.5
15	0.66	0.66	0.053	12.4

### Shape oscillations

**Figure 7:**
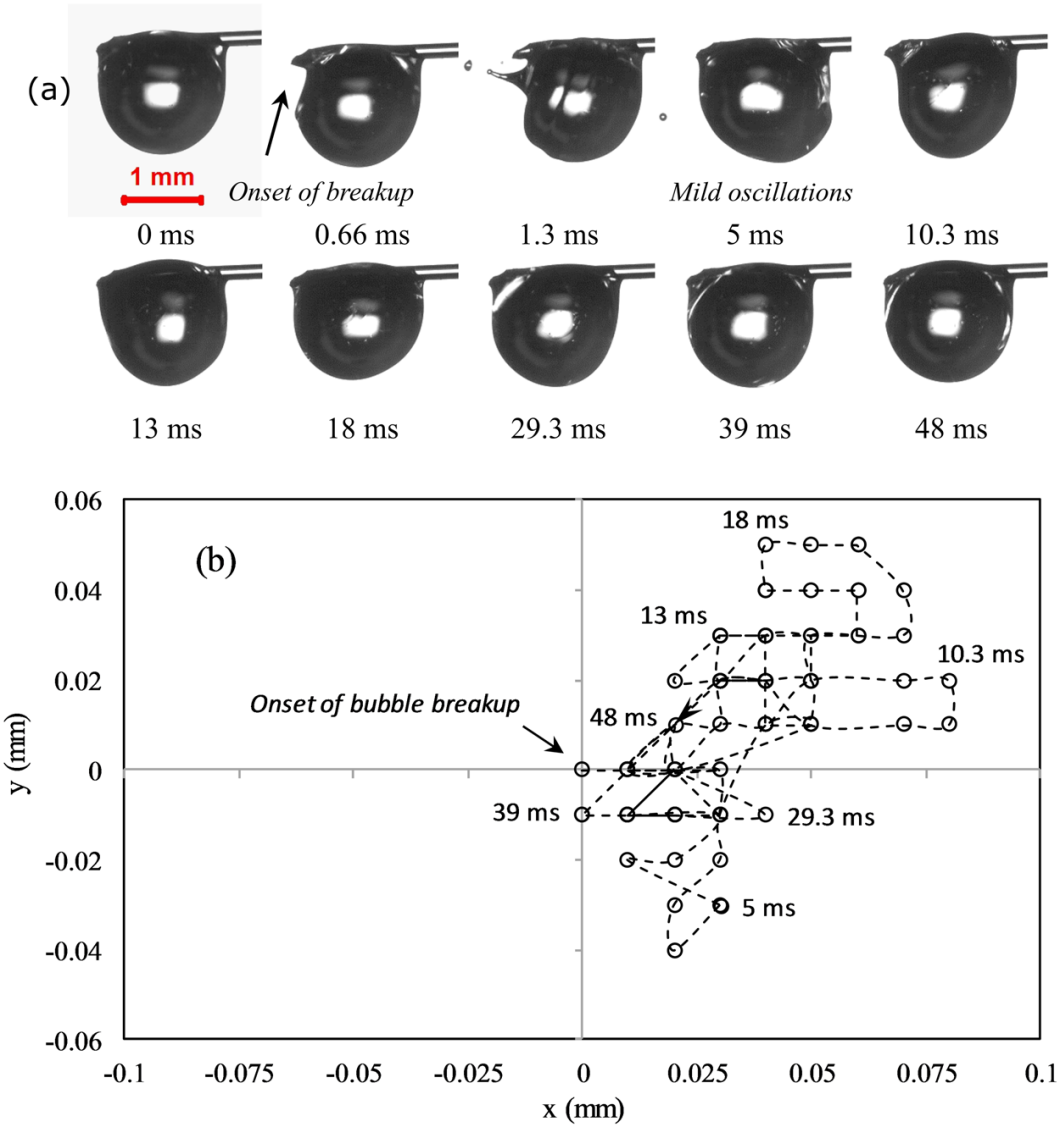
(**a**) Mild oscillations following a minor bubble breakup event (*α* = 0.02) in a typical droplet with a low concentration of volatile component (B30) and (**b**) the trajectory of the droplet centroid following the onset of minor bubble breakup, indicating moderate oscillations of the droplet.

Figure 7a shows a typical minor bubble breakup event, which induces small scale surface oscillations on the parent droplet. The droplet recuperates from the mild oscillations within a short time (~50 ms). The centroid trajectory of the parent droplet following the onset of minor bubble breakup is shown in Fig. 7b. Upon breakup, the droplet centroid follows an orderly trajectory and relocates itself at a distance of 0.025 mm. The maximum spatial displacement in the droplet centroid co-ordinates due to minor breakup events is within ~6% of the initial droplet diameter. On the contrary, the droplet centroid oscillation resulting from the onset of major bubble expulsion event takes a relatively longer time (~100–200 ms) to decay compared to that of minor bubble breakup. Besides, the maximum spatial displacement in the droplet centroid co-ordinates due to major breakup events is within ~18% of the initial droplet diameter.

**Figure 8:**
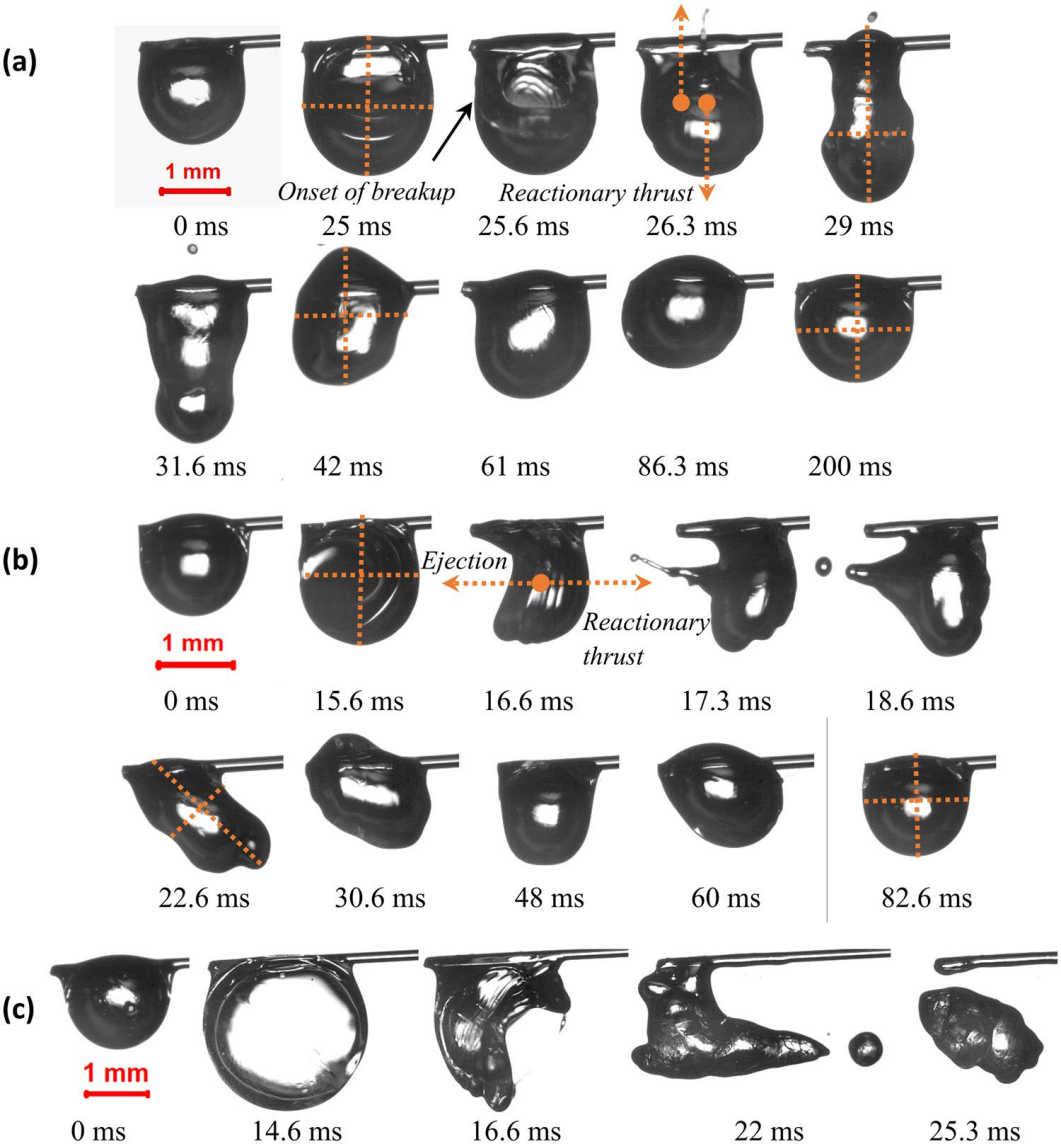
Shape deformations corresponding to the breakup of B30 (*α* = 0.6), B50 (*α* = 0.75), and E50 (α = 3.77) droplets which represent (**a**) bubble breakup at the center of the droplet, (**b**) edge of the droplet and (**c**) micro-explosion.

This high-intensity major bubble breakup causes violent volumetric shape distortions and leads to large scale oscillations as the droplet experiences recoil thrust due to the breakup of a relatively large sized bubble. Upon breakup, the ligament is pushed out of the parent droplet because of the recoiling motion of the liquid surface along with the discharge of ethanol/butanol vapor. Because of the strong recoiling motion, the probability of ligament ejection is also high for major breakup events. The breakup results in repetitious axis-switching and subsequent reorientation of the droplet geometry within a time span of about 100 ms. Figure 8a and b show representative high-speed photographs of major bubble rupture events of B50 and E50 droplets, corresponding to *α* values of 0.6 and 0.75.

Despite having a nearly similar pre-breakup bubble diameter, the resulting droplet dynamics is considerably different due to the collapse of the bubble at distinct sites and depths. Subsequent to both minor and major bubble ruptures, the centroid displacement in the horizontal axis and the vertical axis is dependent on the location of bubble collapse. As seen in Fig. 8a, since the butanol vapor bubble at the pre-breakup instant is sufficiently inside the parent droplet, the breakup results in the expulsion of fewer and considerably smaller sized secondary droplets [O (0.1 mm)].

**Figure 9:**
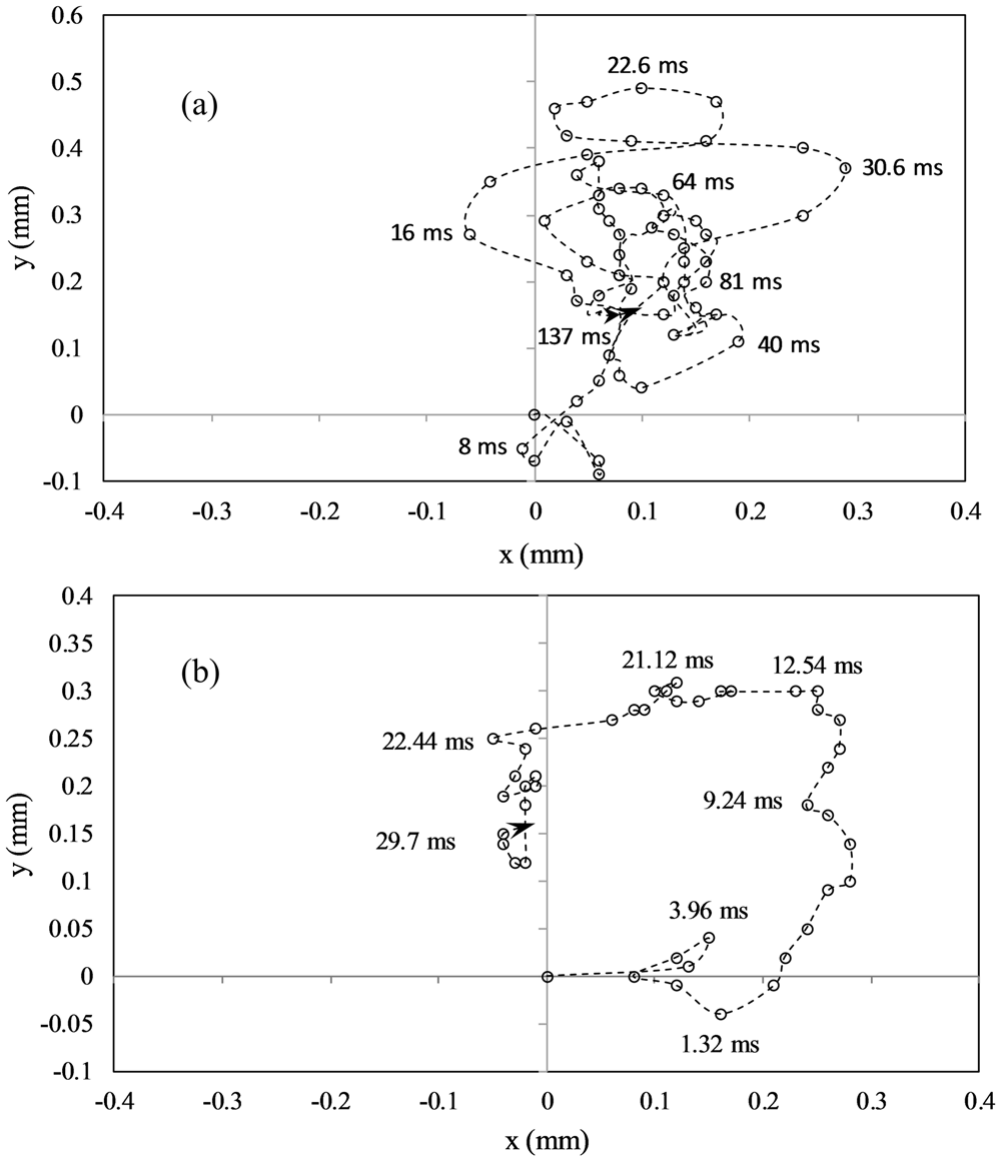
The trajectory of the droplet centroid following the onset of major bubble breakup at (**a**) center of the parent droplet (*α* = 0.6) and (**b**) edge of the droplet (*α* = 0.75), indicating severe oscillations of the droplet.

Moreover, it can be contemplated that mostly gaseous butanol vapor is ejected while the liquid fragmentation is marginal. In contrast, when the bubble ruptures near the surface of the droplet, several droplets are expelled due to the tearing of the liquid layer into fragments. Besides, the average size of secondary droplets is also larger [O (0.2 mm)] as evident in Fig. 8b. The random trajectory of the droplet centroid following the onset of major bubble breakup is shown in Fig. 9a and b, which relate to the sequence of images shown in Fig. 8a and b. The difference in the trajectory is due to the breakup of bubbles at distinct sites. For both butanol/Jet A-1 and ethanol/Jet A-1 blends, 50/50 composition is the optimum proportion for micro-explosion (maximum deformation), resulting in the ejection of significantly larger secondary droplets [O (0.4 mm)]. Thicker ligaments are formed after micro-explosion (Fig. 8c). Unlike other modes of atomization, the ligament possesses significantly small momentum.

**Figure 10:**
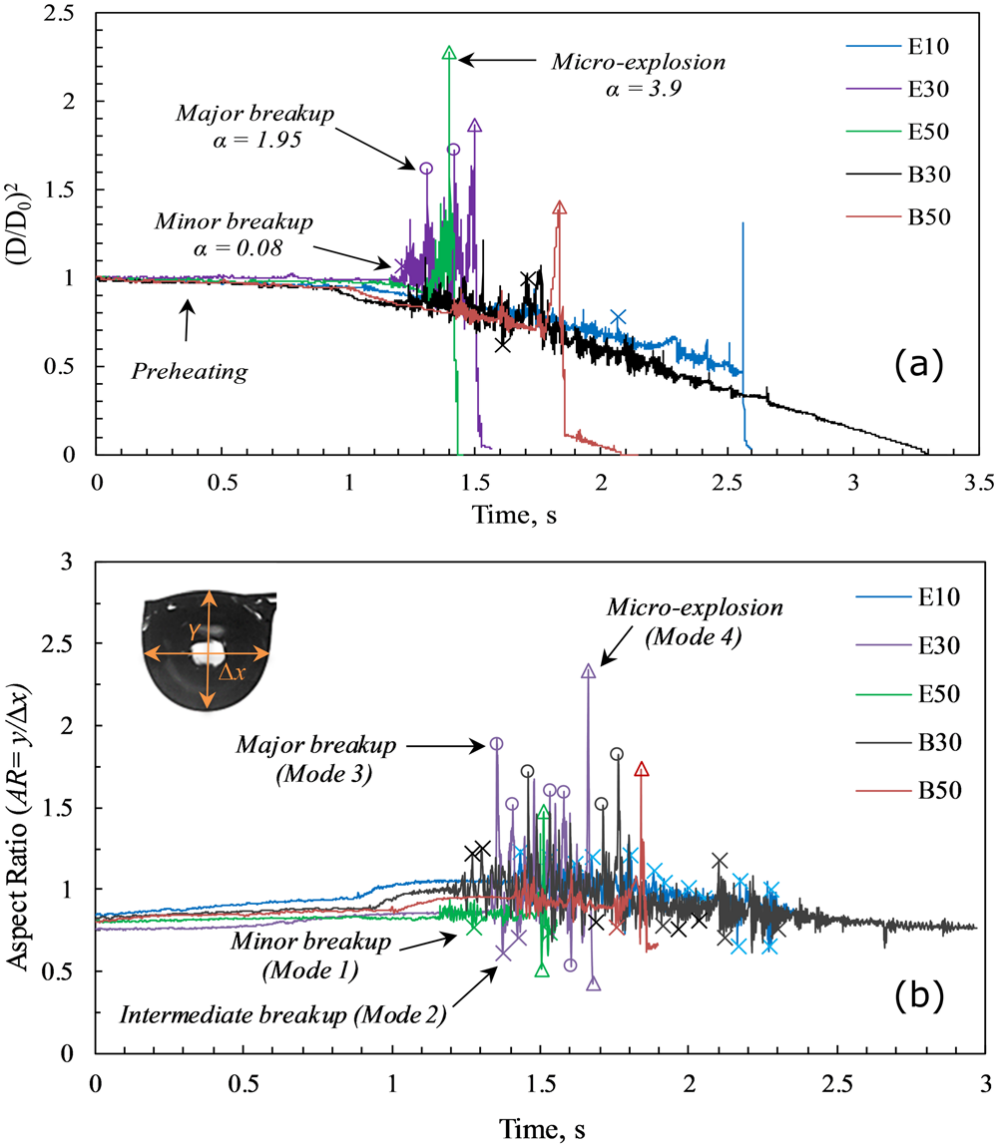
(**a**) Temporal evolution of droplet diameters and (**b**) Temporal evolution of aspect ratio fluctuations of ethanol and butanol blends. Circles indicate high-intensity major bubble breakups. Cross markers indicate minor and intermediate bubble breakups and triangle markers indicate micro-explosions.

Figure 10a represents the temporal evolution of droplet diameter of the blends, which undergo disruption during combustion. The probability of major bubble breakup and micro-explosion events increases as the proportion of higher volatile component is increased in the blends. These features are highlighted in Fig. 10a, which indicates that α increases with time. Major bubble breakups and micro-explosions are clearly identified with the spikes. It can be noticed that large α leads to shorter droplet lifetime signifying complete disintegration of the droplet due to micro-explosion. Furthermore, as *α* increases with time (since the bubble diameter grows continuously with time due to the turbulent mixing), volumetric shape oscillations also increase. The intensity of the bubble breakup events is also represented through the temporal evolution of droplet aspect ratio (*AR* = *y/∆x*) in Fig. 10b. As expected, the aspect ratio fluctuations of droplets increase with an increase in the proportion of higher volatile component for both butanol and ethanol blends. When the percentage of the volatile component is lower, the bubble size at the pre-breakup instant is usually smaller due to inadequate number of nucleation sites. The collapse of this minor bubble leads to the quick detachment of the secondary droplets from the parent droplet. Therefore, the degree of breakup and the resulting variations in the droplet aspect ratio is limited. Moreover, when the vapor bubble develops near the surface of parent droplet, the interface soon ruptures resulting in the discharge of liquid fragments and ethanol/butanol vapor. A large fraction of volatile component results in more nucleation sites consequently forming comparatively larger bubbles. The bubble usually grows at the center of the droplet for a relatively long time. When this bubble breaks apart, the reactionary thrust leads to significant fluctuations in the aspect ratio of parent droplet leading to disintegration. The lower amplitude spikes in Fig. 10b represent the shape deformation in the droplet due to minor and intermediate breakup events, whereas the higher amplitude transient spikes indicate the maximum droplet diameter before major breakup or micro-explosion occurs. It is also evident that the ethanol droplets undergo significantly higher shape deformation compared to butanol blends due to the significant volatility difference between the fuel constituents.

### Interfacial instability

The volatility differential between the fuel components dictates the rate of bubble growth, which in turn controls the degree of droplet disintegration. When the bubble expansion rate is higher, the bubble finds it easier to push the adjacent liquid until it tears apart the parent droplet, whereas the penetration of bubble into the liquid becomes difficult with lower growth rate. The aspect ratio of the parent droplet and the distribution of liquid at the pre-breakup instant were observed to influence the atomization of parent droplet.

**Figure 11:**
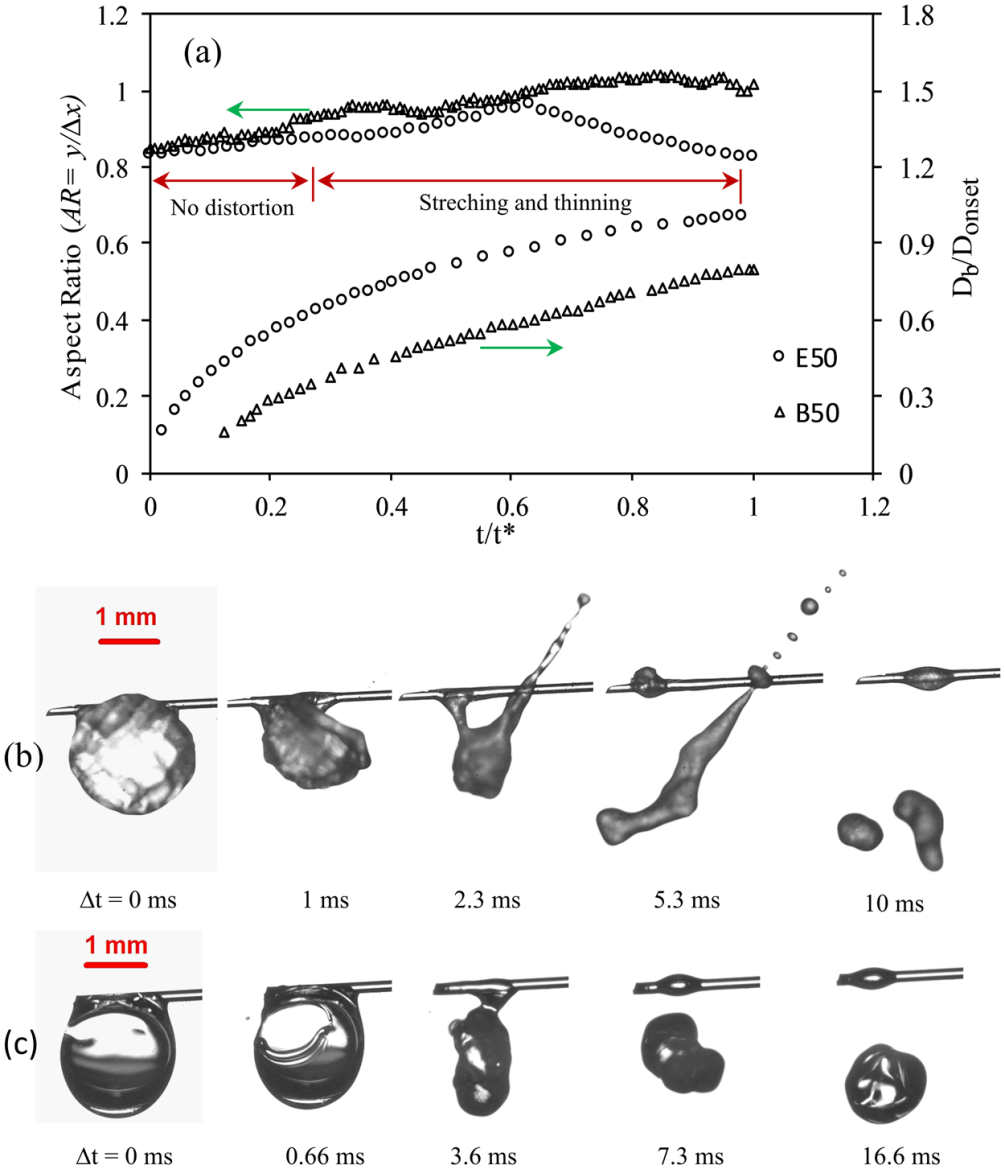
(**a**) Temporal evolution of droplet aspect ratio and bubble diameter during the bubble growth in E50 and B50 blended droplets. The typical breakup of (**b**) E50 droplet with low aspect ratio (AR = 0.95) and (**c**) B50 droplet with high aspect ratio (AR = 1.08).

Figure 11a shows the variation of droplet aspect ratio (during bubble growth) and bubble diameter (D_b_) for E50 and B50 droplets against normalized time (t/t^*^), where the time is normalized with the total bubble lifetime.

The higher aspect ratio of the B50 droplets at the pre-breakup instant (Fig. 11a) can be associated with the moderately slower growth of vapor bubble due to which the liquid layer surrounding the vapor bubble is not uniform. On the contrary, the thickness of the liquid layer at the onset of breakup is nearly uniform in E50 droplet. An approximately even spreading of liquid layer at the pre-breakup instant leads to better atomization of droplet contrary to the non-uniform distribution in B50 droplet (Fig. 11b and c). In fuel blends with significantly larger volatility differential, the rapid expansion of vapor bubble due to the internal pressure forces is also followed by developing surface perturbation^45^. Apart from stretching and thinning of parent droplet due to bubble expansion, an interfacial instability is observed to occur at the vapor-liquid interface in the diffusion controlled stage of bubble growth. The initiation of these perturbations is believed to be the manifestation of Rayleigh-Taylor (RT) instability, which arises when an interface amidst two fluids encounters a pressure gradient opposing the density gradient. Similar perturbations were reported in the numerical work of Elgowainy *et al*.^45^ in emulsified fuels. RT instability can occur when the heavy fluid (liquid) rests above the light fluid (vapor), or when the light fluid is accelerated into the heavy fluid (liquid). During bubble growth, when the vapor embryo grows adequately large and exceeds the equilibrium diameter, there is considerable overpressure in the bubble, which nearly equals the ambient pressure. At this instant, the higher inward acceleration of liquid makes the vapor bubble unstable. If the vapor-liquid interface is perturbed with a small amplitude, then the subsequent disturbances initiate oscillations, further leading to disintegration of the bubble and the parent droplet. In the case of fuel blends with considerably large volatility difference (E50), both the bubble growth and the development of interfacial instability contribute towards the liquid layer thinning, resulting in the breakup of the droplet. Moreover, the rapid bubble growth results in a faster instability growth rate and earlier breakup. The growth rate of the interfacial instability is reduced when the bubble growth is slower since surface tension and viscosity of liquid delays the bubble growth and instability development. In the case of B50 droplets, the vapor bubble continuously expands until the liquid layer is sufficiently thin to break apart, primarily due to stretching. Therefore, for B50 droplets, the droplet breaks up only due to the internal bubble growth. However, there is an additional contribution from the growth of instability resulting in thinning of the liquid layer for E50 blended droplets due to significant differences between the boiling temperatures of constituents. The layer thinning process and the subsequent instability development are the predominant reasons for the relatively prompt breakup and enhanced atomization of droplet as seen in Fig. 11b. This indicates that every droplet, which undergoes micro-explosion, does not necessarily enhance atomization.

**Figure 12:**
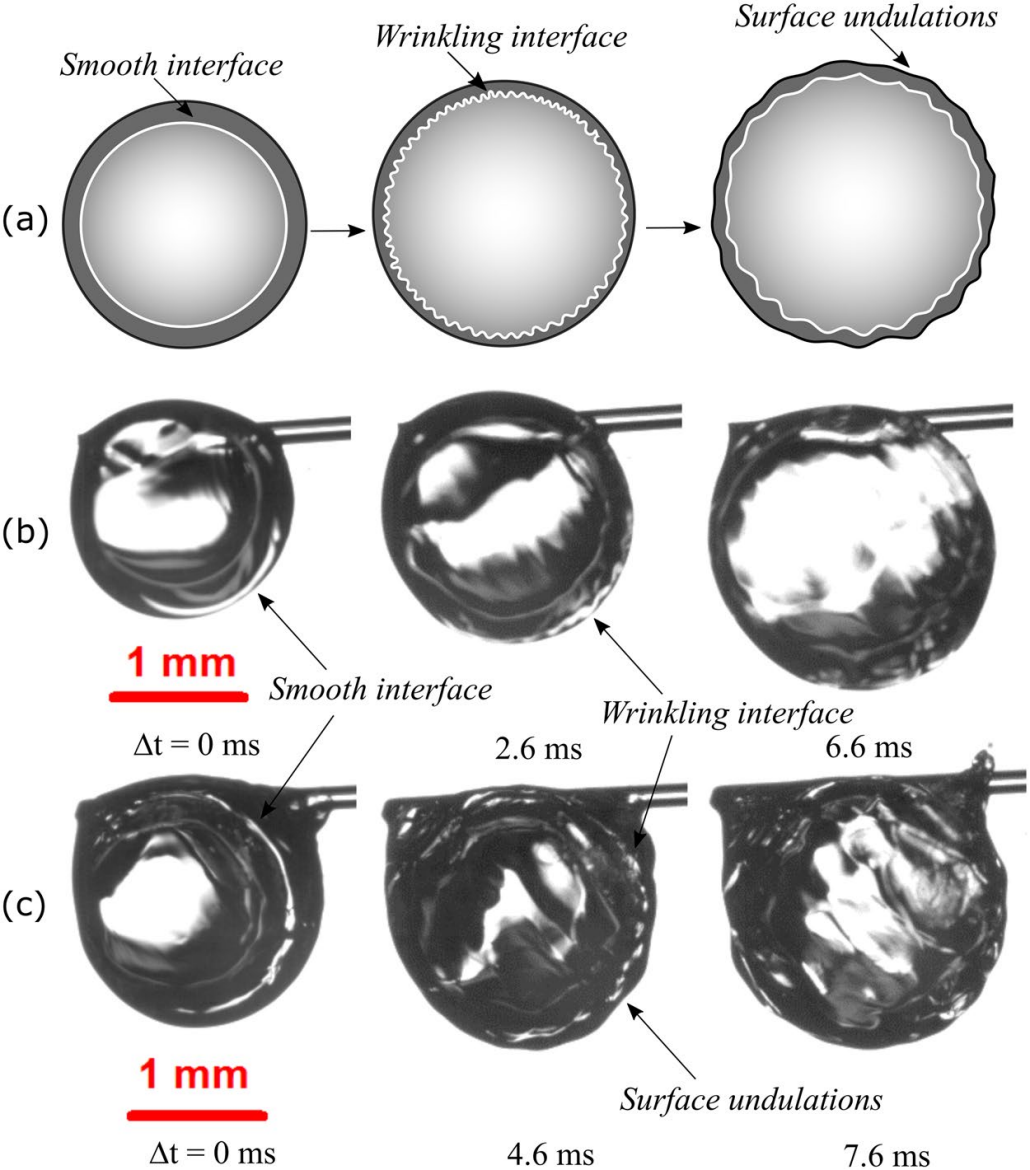
(**a**) Illustration of the interfacial instability in fuel blends. Rayleigh-Taylor instability at the vapor-liquid interface of (**b**) B50 (low probability) and (**c**) E50 (high probability) blended droplets.

Figure 12a illustrates the development of perturbations at the vapor-liquid interface of a multi-component fuel droplet. Figure 12b and c depict wrinkling pattern on the vapor-liquid boundary of B50 droplet and E50 droplet respectively, where ∆t = 0 ms denotes onset of instability.

This wrinkling feature is found to be less probable in fuel blend with relatively small volatility differential (butanol/Jet A-1) where the bubble surface is usually smooth and free from instability throughout its lifetime. The slow bubble growth in butanol/Jet A-1 blends along with higher surface tension and viscosity are possibly the reasons behind the unlikeliness of RT instability. The wrinkling pattern becomes more irregular as the volatility difference is increased between the fuel components. The rapid bubble growth allows the vapor bubble to have a pressure equivalent to ambient pressure such that the heavier liquid on the lighter bubble becomes unstable. The presence of heavier liquid on a vapor initiates small scale roughening of the vapor-liquid interface which further tends to grow until the droplet breaks apart. The length scale of the pattern is observed to be in the range of 85–200 µm. The average instability duration for E50 droplets is around 7 ms (30% of average bubble lifetime). The wavelength of the surface undulation on the droplet due to RT instability varies from 250 µm to 600 µm. An estimation of the RT wavelength was performed on the interface based on the wave instability theory^39^. The RT wavelength, *λ*_*RT*_ can be estimated as follows:

4 $${\lambda }_{RT}=2\pi \sqrt{\frac{3{\sigma }_{L}}{a({\rho }_{L}-{\rho }_{G})}},$$

where σ_*L*_ and *ρ*_*L*_ correspond to surface tension and density of fuel blends and *ρ*_*G*_ represents vapor density of ethanol. The accelerations (*a*) at the vapor-liquid boundary are computed from the sequence of images and are observed to be varying in the order 10^3^–10^4^ times the acceleration of gravity. The wavelength of the disturbance at interface induced by RT instability is 130 µm to 420 µm. The stability theory prediction of the wavelength of the interfacial wrinkling pattern is comparable with the experimental measurements.

## Conclusions

**Figure 13:**
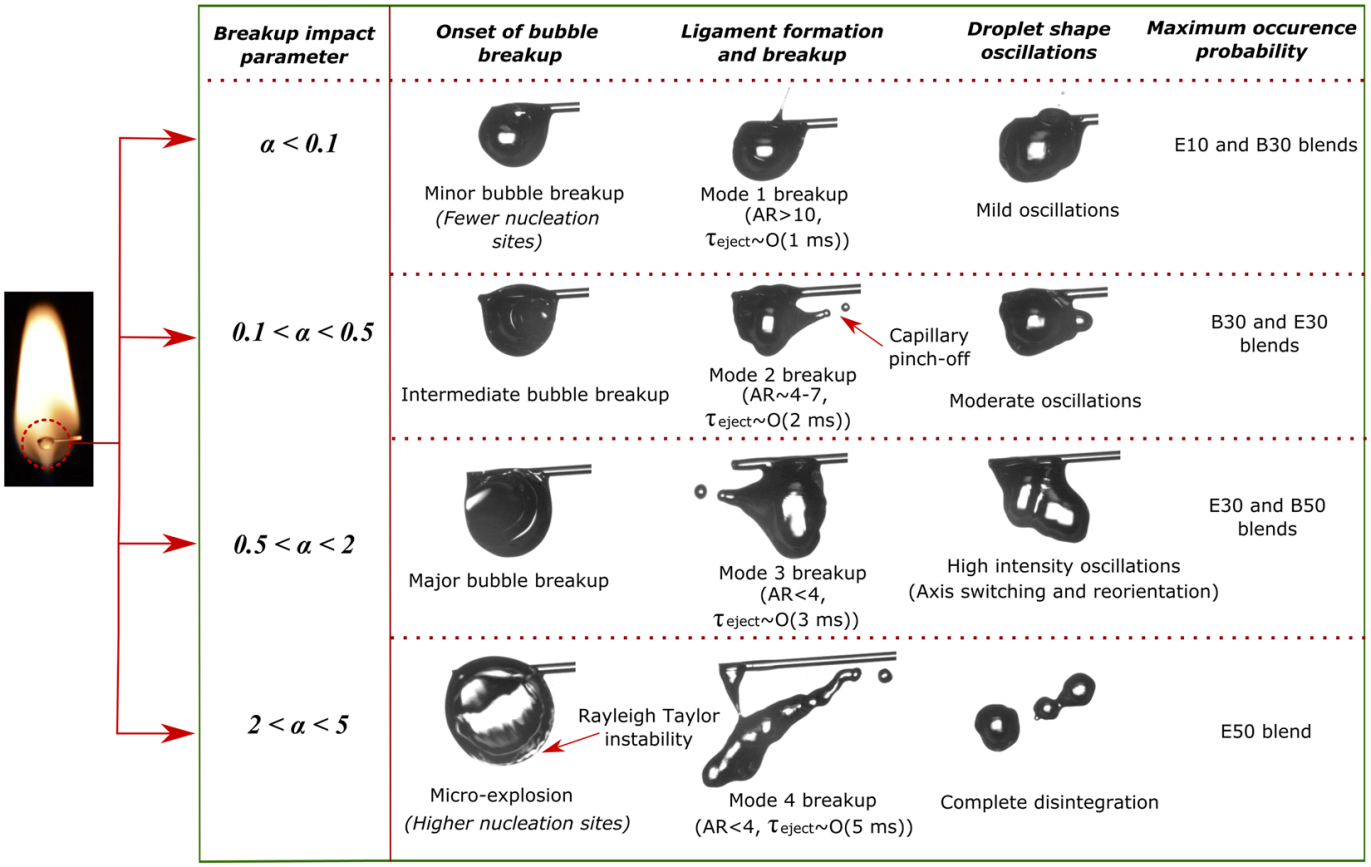
Schematic representing the summary of results.

We studied different modes of ligament breakup and the subsequent shape oscillations of the parent droplet in the combustion of multi-component fuel droplets. Depending on the thickness of ligament, they were categorized into four modes. The breakup time of the ligaments was then compared with the capillary time for all the modes. We observed that minor bubble rupture events induce mild oscillations indicating breakup of high aspect ratio (needle type) ligaments and a major bubble breakup event indicates breakup of low aspect ratio (thick) ligaments which induces severe volumetric shape oscillations. In addition, the parent droplet may not recover from the high degree of deformation caused by micro-explosion. We showed that despite having a similar pre-breakup bubble diameter, the resulting droplet dynamics is considerably different due to the breakup of the bubble at distinct sites and depths. The aspect ratio fluctuations of droplets increase with the increase in the fraction of volatile constituent for both butanol/Jet A-1 and ethanol/Jet A-1 blends. The aspect ratio of the parent droplet and the distribution of liquid at the pre-breakup instance influence the atomization of parent droplet. Figure 13 shows the general summary representing the different regimes of atomization and the underlying instabilities for multiple fuel blends. Finally, we reported the observation of wrinkling pattern (Rayleigh-Taylor instability) at the vapor-liquid interface. This wrinkling is less probable in butanol/Jet A-1 blends and becomes more irregular for ethanol/Jet A-1 droplets. The length scale of the pattern is comparable with that of the wave instability theory. The breakup characteristics and instabilities in the fuel blends studied in this work have the potential to enhance the fuel atomization and reduce the emissions in a spray combustor.

## Materials and Methods

The fuel droplets were burned in a cylindrical chamber at atmospheric pressure and normal gravity. The chamber consists of two quartz windows for optical access (Fig. 1a). A volume-calibrated micropipette was used to create constant volume droplets of 2 ± 0.05 µl of equivalent diameter 1.7 ± 0.1 mm. The droplets were suspended on a 0.2 mm diameter quartz fiber, which is chosen because of its lower thermal conductivity (1.4 W/m K). In the present study, Jet A-1 is used as a base fuel (low volatility) while ethanol and butanol are selected as the high volatility additives. The important physiochemical properties of the selected fuels are summarized in Table 2. Three combinations of ethanol/Jet A-1 and butanol/Jet A-1 are considered in the present work viz; (a) 10% by volume ethanol/butanol in Jet fuel (E10/B10), (b) 30% by volume ethanol/butanol in Jet A-1 (E30/B30), and (c) 50% by volume ethanol/butanol in Jet A-1 (E50/B50). The suspended droplets were heated and ignited using a coiled nichrome wire, which was retracted soon after the droplet was ignited. A high-speed monochrome camera (Phantom V7.3) was used to record the droplet burning process. A reverse mount technique was used to obtain the images of burning droplets with high magnification. The camera resolution and frame rate were maintained at 800 × 600 pixels and 3,000 fps respectively.

**Table 2:** Properties of the tested fuels.

Physical Properties	Jet A-1 (Standard)	Ethanol^50–53^	Butanol^50–53^
Molecular Formula	C_8_–C_16_	C_2_H_5_OH	C_4_H_10_O
Boiling point (°C)	180–250	78.4	117.7
Reid vapor pressure (kPa)	<1	16	2.2
Density at 25 °C (kg/m^3^)	775–840	785	805
Viscosity at 40 °C (mm^2^/s)	<8	1.08	2.63
Surface tension at 25 °C (mN/m)	25.5	21.74	24.02

A light source consisting of 24 high power LEDs was used to illuminate the fuel droplets. Light diffuser films were added between the light source and droplet to assure uniform spatial illumination. An image analysis platform (Image-Pro Plus) was employed to determine the growth of vapor bubble along with the diameter and velocity of secondary droplets. The ambiguity in the measurement of bubble diameter due to the non-spherical or asymmetric shape of the bubble is ±0.05 mm. To guarantee the precision and repeatability of the measurements, all the blends were studied 25 times.
